# Validating RNAi Phenotypes in *Drosophila* Using a Synthetic RNAi-Resistant Transgene

**DOI:** 10.1371/journal.pone.0070489

**Published:** 2013-08-08

**Authors:** Vincent Jonchere, Daimark Bennett

**Affiliations:** Institute of Integrative Biology, University of Liverpool, Liverpool, Merseyside, United Kingdom; St. Georges University of London, United Kingdom

## Abstract

RNA interference (RNAi) is a powerful and widely used approach to investigate gene function, but a major limitation of the approach is the high incidence of non-specific phenotypes that arise due to off-target effects. We previously showed that RNAi-mediated knock-down of *pico*, which encodes the only member of the MRL family of adapter proteins in *Drosophila*, resulted in reduction in cell number and size leading to reduced tissue growth. In contrast, a recent study reported that *pico* knockdown leads to tissue dysmorphology, pointing to an indirect role for *pico* in the control of wing size. To understand the cause of this disparity we have utilised a synthetic RNAi-resistant transgene, which bears minimal sequence homology to the predicted dsRNA but encodes wild type Pico protein, to reanalyse the RNAi lines used in the two studies. We find that the RNAi lines from different sources exhibit different effects, with one set of lines uniquely resulting in a tissue dysmorphology phenotype when expressed in the developing wing. Importantly, the loss of tissue morphology fails to be complemented by co-overexpression of RNAi-resistant *pico* suggesting that this phenotype is the result of an off-target effect. This highlights the importance of careful validation of RNAi-induced phenotypes, and shows the potential of synthetic transgenes for their experimental validation.

## Introduction

For more than 10 years, silencing of gene expression by RNA interference (RNAi) in *Drosophilia melanogaster* has provided a powerful approach to complement classic mutant studies for assessing loss of gene function *in vitro* and *in vivo*. The recent advent of transgenic libraries of inverted repeat constructs capable of expressing dsRNA for virtually any gene of interest *in vivo* [1], has led to the wide uptake of heritable RNAi technology. The use of the UAS-GAL4 expression system to target dsRNA expression to different cell types or stages of development has facilitated targeted genetic screens and allowed manipulation of gene function in different cellular contexts [2]. However, a major limitation of the approach is the high incidence of non-specific phenotypes that arise due to off-target effects [3], [4]. One way to mitigate the risk of misinterpreting RNAi-induced phenotypes is to use independent dsRNA constructs that target non-overlapping sequences of the same gene: if two or more independent lines produce the same effect one can have more confidence that the resulting phenotype is due to knockdown of the gene of interest. However, conflicting results for multiple dsRNAs targeting the same gene are difficult to interpret in the absence of other information. *In silico* predictions of the potential for off-targets (e.g. [5]) can be indicative in this regard, but a genetic complementation test is the best way to validate specificity of the RNAi-induced phenotype. Complementation experiments with wild type transgenes do not control for specificity because the ectopic mRNA may act by titrating RNAi knockdown of endogenous genes, whether they be on- or off-targets. In mammalian systems, where short siRNA molecules are used to induce RNAi, an effective solution to this problem is to test for rescue of the RNAi-induced phenotype using RNAi-resistant transgenes containing silent mutations in the region targeted by the siRNA [6], [7]. Here we have applied this principle to assess complementation of longer dsRNA molecules (typically around 0.5 kb in length) in *Drosophila* to distinguish between on and off-target effects.

The MRL (Mig-10, RIAM, Lamellipodin) family of proteins has been demonstrated to modulate the actin cytoskeleton in response to extracellular signals to effect changes in cell morphology, adhesion and migration [8], [9]. In addition to these roles, we reported that the *Drosophila* MRL orthologue encoded by *pico*, was required for tissue growth [10]. This was supported, in part, through our observations of the effect of RNAi knockdown of *pico*. For these experiments, we developed flies carrying an inverted repeat construct (*picoRNAi^IR4^*) capable of expressing hairpin dsRNA specific for *pico* under GAL4-UAS control. Expression of this construct with a wing-specific GAL4 driver (*MS1096-GAL4*) led to a reduction of *pico* expression and induced a significant reduction in adult wing area. Furthermore, we found that GFP-labelled *picoRNAi^IR4^* clones in wing imaginal discs were small and had a reduced cell doubling time compared to wild-type control cells, without any change in cell cycle phase and cell density. One aspect of this growth effect of *pico* was re-examined in a recent paper [11]. In contradiction with our previous results [10], it was reported that RNAi targeting of *pico* lines leads to severe tissue dysmorphology rather than a strict growth phenotype in the adult wing [11]. Here we have analysed the source of the apparent disparity and find that RNAi lines from different sources exhibit different phenotypic effects. Unlike the construct we previously reported, the commercially available NIG-Fly RNAi lines 11940R-2 and 11940R-3 (referred to as *picoRNAi^R2^* and *picoRNAi^R3^* hereafter), which are different transgenic insertions of an independent inverted repeat construct, reduce the expression of a number of genes unrelated to *pico*, based on qRT-PCR analysis of predicted off-target genes, and produce a crumpled wing phenotype. Importantly, this phenotype is not rescued by overexpression of an RNAi-resistant *pico* construct, indicating that loss of normal wing morphology caused by overexpression of the NIG-Fly RNAi lines for *pico* is most likely caused by off-target effects.

## Results

### RNAi lines targeting pico display distinct adult wing phenotypes

**Figure 1:**
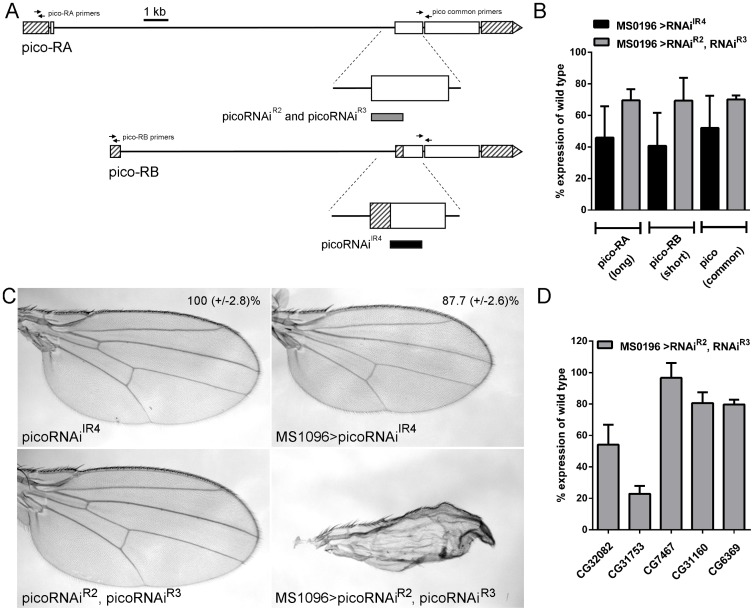
Phenotypic effects of *pico* RNAi constructs. **A**) Gene map showing long (*pico-RA*) and short (*pico-RB*) transcripts. Untranslated regions are shown with hatched boxes, coding exons are shown with open boxes. *pico-RA* and *pico-RB* share identical 3′ exons but differ at their 5′ ends. The position of oligonucleotide primers used to amply either *pico-RA*, *pico-RB* or both *pico* transcripts (for analysis of expression levels shown in panel B) are indicated with arrows. Magnified image of one of the coding exons shows the position of the inverted repeat constructs: picoRNAi^R2^/picoRNAi^R3^ (grey rectangle), picoRNAi^IR4^ (black rectangle). **B**) Ectopic expression of inverted repeat contructs using *MS1096-GAL4* results in knockdown of *pico* mRNA levels in the wing imaginal disc. RNA was extracted from wing imaginal discs that had been dissected from larvae expressing *UAS-picoRNAi^IR4^* (MS1096>picoRNAi^IR4^), or *UAS-picoRNAi^R2^ and UAS-picoRNAi^R3^* (MS1096>picoRNAi^R2, R3^), and was analysed by qRT-PCR. Levels of *picoRA*, *picoRB* and all *pico* transcripts are shown as a percentage of the expression in wing discs from a control strain (*w^1118^*). **C**) Phenotypic effect of ectopic inverted repeat contructs on wing development. Flies carrying UAS-inverted repeat constructs alone (*picoRNAi^IR4^* or *picoRNAi^R2^* and *picoRNAi^R3^*) resemble wild type wings. Ectopic overexpression of *picoRNAi^IR4^* (*MS1096>picoRNAi^IR4^*) resulted in a significant reduction of adult wings size (p<0.001) without any loss of morphology. In contrast ectopic *picoRNAi^R2^*, *picoRNAi^R3^* (MS1096>*picoRNAi^R2^*, *picoRNAi^R3^*) resulted in severe loss of wing morphology as evidenced by crumpled wings. **D**) Ectopic co-overexpression of picoRNAi^R2^ and picoRNAi^R3^ using *MS1096-GAL4* results in the knockdown of at least 4 predicted off-targets as determined by qRT-PCR. Levels of the predicted off targets (*CG32082*, *CG31753*, *CG7467*, *CG31160*, *CG6369*) in *MS1096>picoRNAi^R2,R3^* wing discs are shown as a percentage of the expression in wing discs from a control strain (*w^1118^*).

Previous studies on the function of *pico* loss-of-function have utilised two different sources of inverted repeat constructs to generate loss-of function phenotypes by RNAi-mediated knockdown: *picoRNAi* ^*IR4*^ [10], and *picoRNAi* ^*R2*^ */picoRNAi* ^*R3*^ (NIG-FLY). Mapping of the inverted repeat sequences to the *pico* transcription unit reveals that *picoRNAi* ^*IR4*^ and *picoRNAi* ^*R2,R3*^ map to an overlapping region of long and short *pico* transcripts, (*picoRA* and *picoRB* respectively, Fig. 1A).

To quantify the effect of these RNAi constructs on *pico* expression, we determined the levels of *pico* mRNA in whole wing imaginal discs from 3^rd^ instar larvae either ectopically expressing *picoRNAi^IR4^* or co-expressing *picoRNAi^R2^* and *picoRNAi^R3^* using *MS1096-GAL4* by qRT-PCR (Fig. 1B). In early wondering third-instar wing discs, *MS1096-GAL4* confers transgene expression evenly in both dorsal and ventral compartments of the wing pouch before becoming predominantly expressed in the dorsal half of the pouch. Although *MS1096-GAL4* is not expressed throughout the entire wing disc, ectopic expression of one copy of *picoRNAi^IR4^* using this driver (*MS1096>picoRNAi^IR4^*) resulted in a 52.1% reduction in *picoRA* and *picoRB* mRNA levels in whole wing disc extracts. Co-expression of one copy of both *picoRNAi^R2^* and *picoRNAi^R3^* (*MS1096>picoRNAi^R2,R3^*) also induced a strong depletion of both transcripts, although the knockdown was less pronounced than for *picoRNAi^IR4^*.

Next we assessed the phenotypic effect of *pico* RNAi using a read-out of tissue growth and morphology in the adult wing. *picoRNAi^IR4^*-mediated knockdown was accompanied by a significant reduction (p-value<0.001) of the adult wing size 87.7 +/−2.6%, without any noticeable wing dysmorphology (Fig. 1C), as previously reported [10]. In contrast, *MS1096>picoRNAi^R2,R3^* induced a morphological defect in the adult wings, clearly visible from their crumpled appearance, despite a relatively lower efficacy of *pico* knockdown relative to *picoRNAi^IR4^*. Taken together, these data showed that although the two inverted repeat constructs knock down *pico* expression they exhibit very different phenotypes when ectopically expressed in the developing wing.

### Ectopic pico^R2, R3^ knocks down the mRNA levels of several predicted off targets

To understand why *picoRNAi^R2,R3^* affected tissue morphology, unlike *picoRNAiI^R4^*, we analysed the potential for off-target effects for each RNAi constructs using dsCheck (http://dscheck.rnai.jp/; [5]. Sequence analysis revealed that short inhibitory RNAs (siRNAs) generated from *picoRNAi^IR4^* are highly specific to *pico* transcripts: 0 off-targets from 459 possible mers. In contrast, *picoRNAi^R2,R3^* is predicted to generate siRNA oligomers with perfect homology to 19 other genes (Table 1). To experimentally assess the effect of *picoRNAi^R2,R3^* on the expression of predicted off-target genes, we quantified the relative expression level of the first 5 predicted off-targets in wing discs by qRT-PCR. Notably, we observed a significant decrease in expression (20–81% of wild type, Fig. 1D) for 4 of the 5 off-targets in *MS1096*>*picoRNAi^R2,R3^* discs compared to control discs (*MS1096-GAL4* alone), in agreement with dsCheck predictions.

**Table 1:** List of potential off-targets for *pico* inverted repeat constructs.

picoRNAi^IR4^					picoRNAi^R2^ and picoRNAi^R3^				
mis = 0	mis = 1	mis = 2	FlyBase ID	CG number	mis = 0	mis = 1	mis = 2	FlyBase ID	CG number
459	0	0	FBgn0261811	CG11940-PB	482	4	61	FBgn0261811	CG11940-PA
459	0	0	FBgn0261811	CG11940-PA	339	5	48	FBgn0261811	CG11940-PB
0	2	7	FBgn0022787	CG4261-PA	5	6	30	FBgn0052082	CG32082-PA
0	2	3	FBgn0031988	CG8668-PA	5	6	20	FBgn0045852	CG31753-PA
0	2	2	FBgn0027866	CG9776-PA	5	4	28	FBgn0261885	CG7467-PB
0	2	2	FBgn0021760	CG32435-PA	5	4	28	FBgn0261885	CG7467-PA
0	2	2	FBgn0033460	CG1472-PA	5	4	27	FBgn0261885	CG7467-PC
0	2	2	FBgn0021760	CG32435-PB	5	2	16	FBgn0085405	CG31160-PA
0	2	2	FBgn0021760	CG32435-PC	5	2	2	FBgn0039260	CG6369-PA
0	2	2	FBgn0027866	CG9776-PB	4	5	7	FBgn0264895	CG6682-PA
0	1	7	FBgn0052251	CG32251-PA	4	2	7	FBgn0004889	CG6235-PC
0	1	5	FBgn0031116	CG1695-PB	4	2	7	FBgn0004889	CG6235-PE
0	1	5	FBgn0031116	CG1695-PA	4	2	7	FBgn0004889	CG6235-PD
0	1	5	FBgn0263289	CG5462-PA	4	2	7	FBgn0004889	CG6235-PH
0	1	5	FBgn0263289	CG5462-PD	4	2	7	FBgn0004889	CG6235-PB
0	1	5	FBgn0263289	CG5462-PB	4	2	7	FBgn0004889	CG6235-PG
0	1	5	FBgn0263289	CG5462-PC	3	20	64	FBgn0037252	CG14650-PA
0	1	3	FBgn0033558	CG12344-PA	3	6	32	FBgn0026718	CG17608-PA
0	1	3	FBgn0039554	CG5003-PA	3	3	10	FBgn0038412	CG6898-PA
0	1	3	FBgn0261388	CG15720-PA	3	2	11	FBgn0052333	CG32333-PA
0	1	3	FBgn0053519	CG30175-PA	3	2	5	FBgn0263144	CG8276-PA
0	1	3	FBgn0031299	CG4629-PC	3	2	5	FBgn0263144	CG8276-PB
0	1	3	FBgn0028474	CG4119-PA	2	21	45	FBgn0034072	CG18250-PC
0	1	3	FBgn0024329	CG7717-PA	2	7	14	FBgn0037503	CG14598-PA
0	1	3	FBgn0024329	CG7717-PB	2	4	13	FBgn0261938	CG4644-PA
0	1	3	FBgn0031299	CG4629-PB	2	4	3	FBgn0028380	CG9670-PA
0	1	3	FBgn0031299	CG4629-PA	2	3	14	FBgn0004435	CG17759-PA
0	1	2	FBgn0051301	CG31301-PA	2	3	14	FBgn0004435	CG17759-PG
0	1	2	FBgn0028647	CG11902-PA	2	3	8	FBgn0263396	CG16901
0	1	2	FBgn0037391	CG2017-PB	1	22	117	FBgn0016694	CG17888-PD

Off-targets were determined using dscheck (http://dscheck.rnai.jp/), which generates all possible 19 mers that can be theoretically generated from a longer dsRNA sequence and determines their homology to *Drosophila* genes identified by their Flybase ID and Celera Genomics (CG) number. The number of 19 mers matching each gene found by the sequence comparison are tabulated, where Mis = 0, Mis = 1, Mis = 2 correspond to the number of 19 mers with 0, 1 and 2 mismatches for each gene, respectively.

### Wing dysmorphology phenotypes of picoRNAi^R2,R3^ are not rescued by over-expression of an RNAi-resistant pico transgene

**Figure 2:**
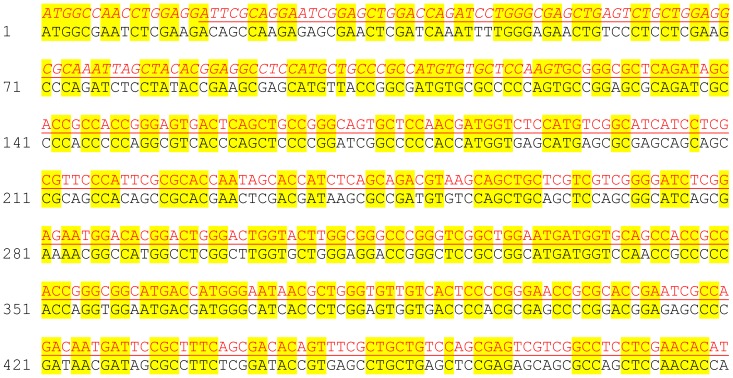
Sequence comparison of wild type and synthetic *pico* genes. Shown is a sequence alignment of the first 493 bp of the synthetic *pico* transgene (in black) and the equivalent region of the endogenous *pico* gene (in red). Identical bases in the two sequences are highlighted in yellow. The regions corresponding to picoRNAi^IR4^ is underlined. The region overlapping with that of picoRNAi^R2/R3^ is in italics.

**Figure 3:**
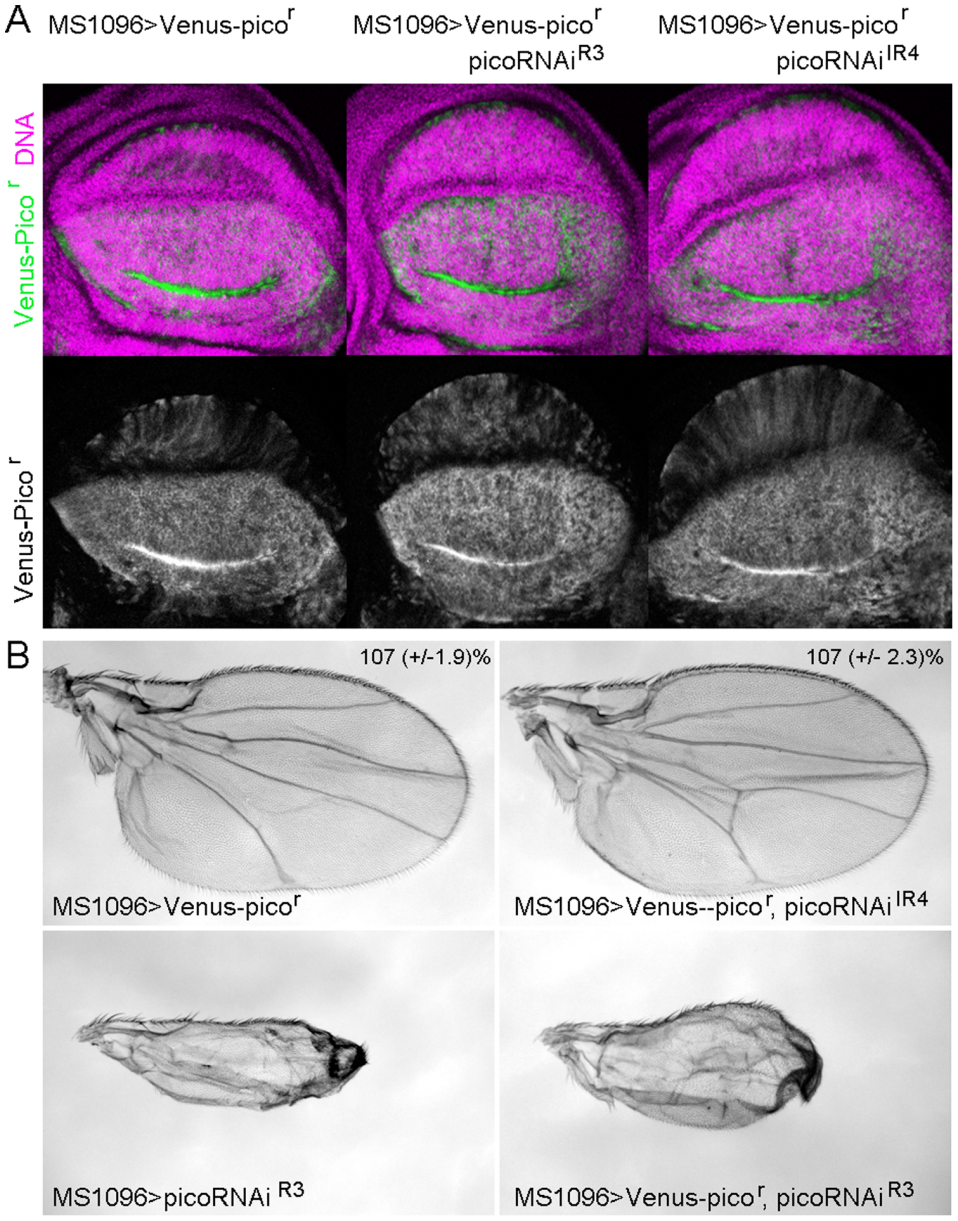
A synthetic RNAi-resistant *pico* transgene rescues the phenotypic effect of ectopic picoRNAi^IR4^ but not picoRNAi^R3^. A) Expression of a synthetic, Venus-tagged, *pico* transgene is not affected by co-expression of *picoRNAi* constructs. Confocal images of wing imaginal discs from flies expressing *UAS-Venus-pico^r^* alone (MS1096>Venus-pico^r^), or together with *UAS-picoRNAi* constructs (MS1096>Venus-pico^r^, *picoRNAi^R3^* or MS1096>Venus-pico^r^, *picoRNAi^ IR4^*) are shown. DNA staining with TO-PRO-3 (magenta in the merged image) reveals that each image is of a similar section through the disc, whilst the Venus signal (green in the merged image) reveals that the levels of Venus-pico^r^ are largely unaffected by co-expression with the pico inverted repeat constructs. B) Ectopic expression of Venus-pico^r^ rescues the growth defect resulting from picoRNAi^IR4^, but not the wing dysmorphology phenotype displayed by picoRNAi^R3^. Ectopic Venus-pico^r^ under the control of MS1096-GAL4 (MS1096>Venus-pico^r^) results in an increase in wing size. Ectopic picoRNAi^R3^ (MS1096> picoRNAi^R3^) results in a crumpled wing (similar to the effect of co-expressing picoRNAi^R2^ and picoRNAi^IR3^, as in Figure 1C), and this is not suppressed by Venus-pico^r^ (MS1096>Venus-pico^r^, picoRNAi^R3^). In contrast, coexpression of Venus-pico^r^ and picoRNAi^IR4^ (MS1096>Venus-pico^r^, picoRNAi^IR3^) resembles the effect of Venus-pico^r^ alone.

To test if the wing dysmorphology phenotype resulting from overexpression of picoRNAi^R2/R3^ was due to off-target effects of the inverted repeat construct, we developed an RNAi-resistant form of *pico* (*pico^r^*) that could be used in genetic complementation tests. This was done by incorporating numerous silent polymorphic mutations into a synthetic gene construct encoding the short isoform of Pico (*picoRB*). Changes in the codon usage that we introduced consequently meant that, in the regions targeted by the RNAi, homology with the inverted repeat sequences was limited to no more than 8 contiguous base pairs (Fig. 2). To assess the resistance of ectopic *pico^r^* to dsRNA for *pico*, we generated transgenic flies capable of expressing Venus-tagged *pico^r^* under UAS-GAL4 control and analysed the levels of the Venus-tagged ectopic protein in the presence or absence of the inverted repeat constructs. Venus, a variant of enhanced Yellow Fluorescent Protein, was readily detectible when the tagged protein was ectopically expressed in the wing pouch under the control of *MS1096-GAL4*. Notably, ectopic expression of Venus-Pico*^r^* was not modified by co-expression of *picoRNAi^IR4^* or *picoRNAi^R3^*, demonstrating that ectopic Pico^r^ is resistant to RNAi-mediated knockdown (Fig. 3A).

Next we tested whether the phenotypic effect of *MS1096>picoRNAi^IR4^* or *picoRNAi^R3^* could be rescued by overexpression of Pico^r^ (Fig. 3B). Previously we reported that overexpression of wild type *pico* short with *MS1096-GAL4* resulted in modest tissue overgrowth. Similarly, ectopic-tagged *Venus-pico^r^* resulted in a 107% increase in adult wing size, indicating that the Venus tag does not interfere with its ability to promote growth. When we coexpressed *pico^r^* together with *picoRNAi^IR4^* the adult wings resembled those of ectopic *pico^r^* alone, indicating that the reduced wing size resulting from *MS1096>picoRNAi^IR4^* was rescued by expression of *pico^r^*. Notably, the effect of *pico^r^* and *picoRNAi^IR4^* was not simply additive, indicating that the level of Pico^r^ is likely to be much more than two fold that of endogenous levels. In contrast, ectopic *pico^r^* failed to rescue the crumpled wing resulting from *picoRNAi^R3^* overexpression (Fig. 3B). Taken together, these data confirm that specific RNAi-mediated knockdown of *pico* results in a tissue growth phenotype, and indicate that the tissue dysmorphology phenotype associated with *picoRNAi^R3^* is attributable to an off-target effect.

## Discussion

Comprehensive validation of an RNAi-induced phenotype requires demonstration that: i) the expression level of the intended target is reduced by the RNAi; ii) the expression of any potential off-targets is not affected; and, iii) the RNAi-induced phenotype can be reversed by expression of the wild type protein; genetic complementation should be with an RNAi-resistant form of the gene, and not rely on overexpression of wild type mRNA that could sequester siRNA molecules from both off- and on-targets alike. Applying these principles to the validation of *pico* RNAi lines, we set about to determine whether the discrepancy in reported phenotypes for pico knockdown could be explained by potential off-target effects. Firstly, we assessed the levels of *pico* mRNA in wing discs expressing *pico* inverted repeat constructs. qRT-PCR revealed that both of the RNAi constructs tested (*picoRNAi^IR4^* and *picoRNAi^R2/R3^*) were effective in knocking down *pico* mRNA levels. The efficacy of *picoRNAi^IR4^* is consistent with our previous observations that levels of ectopically expressed epitope-tagged Pico were reduced in the presence of *picoRNAiI^R4^* [10]. Next, we analysed potential off-targets *in silico* and in extracts. Unlike picoRNAi^IR4^, picoRNAi^R2/R3^ was predicted to affect the expression of numerous off-target genes. qRT-PCR analysis confirmed that the expression level of 4 out of 5 of the predicted off-targets tested were indeed reduced by *picoRNAi^R2/R3^*. Notably, although the number of siRNA oligomers that matched the off-target genes was low (e.g. 5 predicted off-target 19 mers as compared to 482 on-target 19 mers, Table 1), they nevertheless drastically affected the gene expression of these off-target genes. This highlights the importance of testing the specificity of RNAi lines experimentally. To do this, we tested the ability of a synthetic transgene to rescue the RNAi-induced phenotypes. Phenotypic analysis of *picoRNAi^IR4^* and *picoRNAi^R2/R3^* revealed two distinct effects: *picoRNAi^R2,R3^* promoted loss of normal wing morphology, whereas *pico^IR4^* induced a reduction in wing size. Importantly, we found that the effect of *picoRNAi^R3^* was not rescued by overexpression of the RNAi-resistant form of *pico* (*pico^r^*) and is most likely a consequence of the reduced expression of one or more of its predicted off-targets.

Our findings reinforce the value of experimentally testing the specificity of RNAi-induced phenotypes to ensure they are correctly attributed to knockdown of the gene of interest. In general, the strategy that we have employed using synthetic transgenes is widely applicable to the validation of any RNAi construct. Efficient gene synthesis of long stetches of DNA has become increasingly affordable in the last few years and rivals the cost of conventional cloning approaches. Almost any coding region of a gene can be rendered resistant because, on average, every third base can be substituted due to the degeneracy of the genetic code. However, regions containing multiple codons for either Methionine or Tryptophan, which do not allow sequence substitutions if the amino acid sequence is to be maintained, are refractory to this approach. Untranslated regions targeted by an RNAi construct can simply be omitted from any rescue construct [12], [13]. By extension, this approach could be utilised to test the role of critical domains or single amino-acids by engineering the synthetic transgene to harbour additional genetic changes that affect the coding sequence of the ectopic protein. The use of the GAL4-UAS system means that knockdown and complementation experiments can be performed in a tissue-specific or stage specific manner. Furthermore, a range of UAS-expression vectors are publicly available with fluorescent and epitope tags that can be used to readily monitor the ability of the transgene to elude RNAi-mediated knockdown. However, the use of GAL4-UAS system for genetic complementation tests is not without its limitations. Firstly, the use of a GAL4 driver (and to a lesser extent changes to codon usage) means that levels of the synthetic transgene are not endogenous. Secondly, whereas an on-target RNAi should only have an effect in cells where there is endogenous expression of the target gene, the protein produced from a synthetic rescue construct may be ectopically expressed in cells where there is no endogenous expression. This may be mitigated by the use of an enhancer trap GAL4 line driving GAL4 under the control of the target gene promoter, but enhancer trap lines that faithfully replicate the endogenous expression pattern only exist for a minority of genes.

Another strategy that has recently been developed to test genetic complementation in *Drosophila melanogaster* is based on the use of cross-species transgenic constructs [14], [15]. This method uses the genomic DNA of different *Drosophila* species such as *Drosophila pseudoobscura* that are divergent enough from the host sequence to make the genomic constructs RNAi-resistant. However, this approach is also not without its limitations. Cross-species constructs may also show differences in their pattern of expression relative to the endogenous gene in *Drosophila melanogaster*, not least because of position effects associated with the insertion site of the transgene. In addition, the genomes of the donor species often carry non-synonymous substitutions affecting the amino-acid sequences of the gene products. Very minor changes in amino acid sequences can abolish functional rescue in interspecies crosses for example by drastically affecting the ability of proteins from divergent species to interact correctly with one another [16]. In addition, because the constructs are not tagged it is hard to readily assess whether protein levels are refractory to the effects of the RNAi.

In summary, we report here the results of experiments to test the specificity of two RNAi constructs for the *pico* gene that display different phenotypic effects. Notably one of the RNAi constructs affects the expression of various off-target genes despite the prediction that only a limited number of siRNA oligomers generated from the full-length dsRNA target these loci. We find the use of synthetic gene fragments resistant to knockdown by RNAi to be an effective approach to distinguish between specific and non-specific effects.

## Materials and Methods

### Fly husbandry and genetics

Flies were reared at 25°C under standard conditions. *pico^IR2-3^* RNAi lines came from NIG-Fly (National institute of Genetics: 11940R); *pico^IR4^* was described in a previous paper [10]. Phenotypic analysis of adult wings and effect of *pico* RNAi lines on pico mRNA expression was determined using the wing imaginal disc driver *MS1096-GAL4*. Genotypes were as follows:

- 1B, D *MS1096-GAL4*
- 1B, D *MS1096-GAL4; UAS-picoRNAi^R2^/+; UAS-picoRNAi^R3^/+*
- 1B *MS1096-GAL4; UAS-picoRNAi^IR4^/+*
- 1C *UAS-picoRNAi^IR4^/+*
- 1C *MS1096-GAL4/Y; UAS-picoRNAi^IR4^/+*
- 1C *UAS-picoRNAi^R2^/+; UAS-picoRNAi^R3^/+*
- 1C *MS1096-GAL4/Y; UAS-picoRNAi^R2^/+; UAS-picoRNAi^R3^/+*
- 2A,B *MS1096-GAL4/Y; UAS-Venus-pico^r^/+*
- 2A,B *MS1096-GAL4/Y; UAS-Venus-pico^r^/+; UAS-picoRNAi^R3^/+*
- 2A,B *MS1096-GAL4/Y; UAS-Venus-pico^r^/+; UAS-picoRNAi^IR4^/+*
- 2A,B *MS1096-GAL4/Y; UAS-picoRNAi^R3^/+.*

### Wing Area Analysis

Male adult flies wings were dissected and fixed in 75% ethanol, and mounted on glass slides in Canadian Balsam mounting medium (Gary's Magic Mountant) and examined by light microscopy. The wing area, exclusive of the alula and the costal cell, was measured using NIH ImageJ (http://rsb.info.nih.gov/ij/), n = 35 per genotype. To avoid observer bias in the measurements of wing areas, experimenters were blinded to the genotype of flies. A t-test was applied to test for a significant effect of *MS1096-GAL4*, UAS-picoRNAi on wing size by comparison with UAS-picoRNAi alone.

### RNA isolation and qRT-PCR

3rd instar larval imaginal tissues were dissected in cold Phosphate Buffered Saline buffer, put in RNAlater (Invitrogen), quickly frozen in liquid nitrogen and stored at −80°C until isolation of RNA. 3 pools of imaginal discs were made for each condition tested (*MS1096-GAL4*; *MS1096>pico^IR4^*; *MS1096>picoI^R2-3^*) corresponding to at least 24 imaginal discs/pool. RNA extractions were performed using the Ambion RNAqueous-Micro Kit (Invitrogen). RNA concentrations were measured at 260 nm and RNA integrity was evaluated on a 2% agarose gel. 1 µg of total RNA samples were subjected to reverse-transcription using High capacity RNA-to-cDNA kit (Applied biosystems/Invitrogen). Primer design was performed using Primer3 online software, http://frodo.wi.mit.edu/ [17]. The specificity of primers was assessed by sequencing of PCR products (GATC Biotech), and alignment of the resulting sequences by performing BLASTN against the *Drosophila melanogaster* transcriptome. cDNA were amplified in real time using the qPCR Master mix plus for power SYBR Green I assay (Invitrogen) and analysed with the StepOnePlus Real-Time PCR System (Applied Biosystems). Each run included triplicates of control cDNA corresponding to a pool of imaginal discs from *MS1096-GAL4* and *MS1096>picoRNAi* lines, no-template controls and samples. The threshold cycle (Ct) was determined for each sample and control cDNA. A calibration curve was calculated using the Ct values of the control cDNA samples and the relative amounts of unknown samples were deduced from this curve. The level of expression for genes tested with different RNAi lines was compared to wild-type expression (in an *MS1096-GAL4* strain) and expressed as a percentage of the latter.

### RNAi-resistant construct design

Codon usage in the transcript *picoRB* was modified with the introduction of silent mutations to render it resistant to RNAi-mediated knockdown, whilst minimising the use of rare codons that are recognised by low abundance tRNA species. 187/493 bp were changed in the region targeted by *picoRNAi^IR4^* and *picoRNAi^R2,3^* as shown in Fig. 2. *pico^r^* was synthesised and subcloned into pDONR221 Gateway cloning vector (Invitrogen). Gateway LR reactions were performed to shuttle *pico^R^* from the pDONR221 entry vector into pTVW (UASt promoter with an N-terminal Venus tag) vector (*Drosophila* Genomics Resource Center) for expression in flies with an N-terminal Venus Tag. Transgenic flies were generated using *P* element mediated germline transformation of a *w^1118^* strain.
